# Effects of chlorhexidine preprocedural rinse on bacteremia in periodontal patients: a randomized clinical trial

**DOI:** 10.1590/1678-7757-2017-0112

**Published:** 2017

**Authors:** Rodrigo Dalla Pria Balejo, José Roberto Cortelli, Fernando Oliveira Costa, Renata Magalhães Cyrino, Davi Romeiro Aquino, Karina Cogo-Müller, Taís Browne Miranda, Sara Porto Moura, Sheila Cavalca Cortelli

**Affiliations:** 1 Universidade de Taubaté, Departamento de Odontologia, Taubaté, SP, Brasil; 2 Universidade de Taubaté, Departamento de Odontologia, Núcleo de Pesquisa Periodontal, Taubaté, SP, Brasil; 3 Universidade Federal de Minas Gerais, Faculdade de Odontologia, Belo Horizonte, MG, Brasil; 4 Universidade Estadual de Campinas, Faculdade de Ciências Farmacêuticas, Piracicaba, SP, Brasil

**Keywords:** Bacteremia, Periodontal diseases, Periodontal debridement, Mouthwashes

## Abstract

**Objective:**

Single dose of systemic antibiotics and short-term use of mouthwashes reduce bacteremia. However, the effects of a single dose of preprocedural rinse are still controversial. This study evaluated, in periodontally diseased patients, the effects of a pre-procedural mouth rinse on induced bacteremia.

**Material and Methods:**

Systemically healthy individuals with gingivitis (n=27) or periodontitis (n = 27) were randomly allocated through a sealed envelope system to: 0.12% chlorhexidine pre-procedural rinse (13 gingivitis and 13 periodontitis patients) or no rinse before dental scaling (14 gingivitis and 15 periodontitis patients). Periodontal probing depth, clinical attachment level, plaque, and gingival indices were measured and subgingival samples were collected. Blood samples were collected before dental scaling, 2 and 6 minutes after scaling. Total bacterial load and levels of *P. gingivalis* were determined in oral and blood samples by real-time polymerase chain reaction, while aerobic and anaerobic counts were determined by culture in blood samples. The primary outcome was the antimicrobial effect of the pre-procedural rinse. Data was compared by Mann-Whitney and Signal tests (p<0.05).

**Results:**

In all sampling times, polymerase chain reaction revealed higher blood bacterial levels than culture (p<0.0001), while gingivitis patients presented lower bacterial levels in blood than periodontitis patients (p<0.0001). Individuals who experienced bacteremia showed worse mean clinical attachment level (3.4 mm vs. 1.1 mm) and more subgingival bacteria (p<0.005). The pre-procedural rinse did not reduce induced bacteremia.

**Conclusions:**

Bacteremia was influenced by periodontal parameters. In periodontally diseased patients, pre-procedural rinsing showed a discrete effect on bacteremia control.

## Introduction

Periodontal diseases contribute to systemic disorders that involve inflammatory mediators in the bloodstream and the migration of microorganisms and their products throughout the body^14^ ^,^ ^15^. Bacteremia can be induced by simple daily habits such as oral hygiene^14^ ^,^ ^16^ and mastication^6^ ^,^ ^9^ or by more invasive procedures such as dental scaling^13^ ^,^ ^14^ ^,^ ^20^ ^,^ ^30^. The intensity of injury, microbiota profile, severity of inflammation, and local infection affect bacteremia^27^. This partially explains why periodontal diseases contribute to bacteremia development. However, a systematic review indicated that the heterogeneity of high methodological quality studies impaired comparative analysis, leading the authors to note the need for randomized, controlled clinical trials to provide more accurate data about bacteremia in periodontics^13^.

Over the years, oral bacteria have developed mechanisms to invade and persist in the host cells, escape host immune surveillance, adapt to niches at extra-oral sites, and induce inflammatory responses leading to adverse systemic effects. In conjunction, available evidence corroborates the view that periodontitis acts as a biologically plausible risk factor for systemic diseases. In fact, transient bacteremia, systemic injury by free toxins of oral pathogens, and systemic inflammation caused by soluble antigens of oral pathogens have been implicated in the link between oral and systemic conditions. However, there is no clear understanding of the mechanisms of oral bacteria in extra-oral infections and inflammation, which limits effective therapies. Therefore, reduction of the entrance of bacteria and their products in blood stream could represent a reliable health care tool^11^ ^,^ ^21^.

Pre-procedural rinses are used to reduce crossinfection^8^ ^,^ ^10^ ^,^ ^16^, and, based on their antimicrobial properties, it is reasonable to expect a positive effect on bacteremia. However, randomized clinical trials investigating the effects of pre-procedural rinses on bacteremia are scarce.

We hypothesised that susceptible individuals undergoing manual dental scaling develop bacteremia, which could be reduced employing a pre-procedural rinse. Therefore, this study evaluated whether a single dose of pre-procedural mouthrinse in periodontally diseased patients reduces bacteremia stimulated by manual dental scaling. We also analyzed the occurrence and magnitude of bacteremia based on the results from culture and real-time polymerase chain reaction (PCR) techniques.

## Material and methods

### Trial design

This randomized, double-blind, single-center, parallel clinical trial was conducted at the University of Taubaté, Brazil, from August 2014 to December 2014. This study was registered at Clinicaltrials.gov (NCT NCT02215473) and was approved by the Institutional Ethics Committee (protocol 521/10). The pilot study was ethically approved under this same protocol. All participants provided an informed consent form.

### Study population

**Figure 1:**
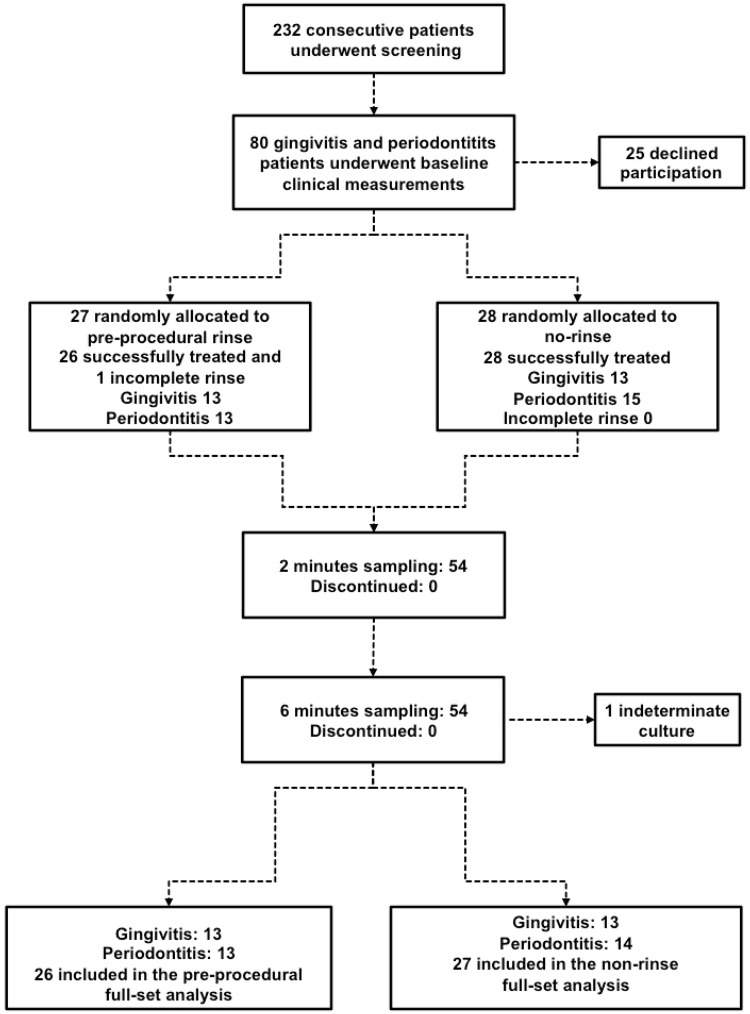
Study design from screening to completion of the 6-month study

Systemically healthy individuals (18 and 45 years) diagnosed with either plaque-related gingivitis^1^ or moderate chronic periodontitis^1^, male or female, with at least 20 natural teeth, with no need for antibiotic prophylaxis, and with clinical indication for dental scaling, composed the study population (Figure 1). A single calibrated (kappa=0.84 for periodontal probing depth - PD and 0.82 for clinical attachment level -CAL) examiner measured PD, CAL, plaque (Pl)^25^, and gingival indices (GI)^17^ at four sites *per* tooth using a manual periodontal probe (PCPUNC, Hu-Friedy, Chicago, IL, USA). One panoramic radiograph was obtained for each patient.

Exclusion criteria were: history of systemic diseases; current antibiotic therapy or antibiotic use in the past 3 months; use of immunosuppressants; regular antiseptic use in the past 3 months; furcation lesions; periodontal treatment 12 months preceding the start of the study; and removable dentures, braces and risk conditions for bacteremia.

The sample size calculation was determined based on a previous study^6^ and adjusted after the pilot study (n = 2 *per* group) that included the processing of realtime PCR. Volunteers from the pilot study did not participate in the main study. The minimum number of 12 individuals per group was designed to provide 80% power and a 5% significance level.

According to periodontal condition, two blocks of patients were randomly allocated to one of two groups by opaque closed and numbered envelopes containing identifications for either the pre-procedural rinse or non-rinse groups (Figure 1).

### Bacteremia induction and periodontal treatment

Two trained periodontists carried out the periodontal treatment. Pre-procedural rinse and non-rinse groups were booked on separate days. At bacteremia induction, the individuals attended in the morning without oral hygiene after 8 hours of no food or drink, except water. Participants in the pre-procedural rinse groups performed a single rinse with 15 mL of 0.12% chlorhexidine (Periogard^®^, Colgate-Palmolive, São Bernardo do Campo, SP, Brazil) for 30 seconds and were immediately anesthetized, and 3 minutes after rinsing the scaling and root planning was performed with Gracey and McCall curettes and Hirschfield-type files. This preliminary scaling aimed at stimulating bacteremia, started in the sampled sites and was extended to the entire dental hemi-arch within 6 minutes. Periodontal treatment was concluded in additional visits according to individual needs.

### Biofilm and blood sampling

One hour prior to the induction of bacteremia, subgingival samples were collected^5^ and stored at −80°C in empty minitubes. Blood samples were collected^6^ at 3 sequential times: before dental scaling (T0), 2 minutes (T1), and 6 minutes (T2) after dental scaling had started. At each time, 2 mL of peripheral blood was collected into citrated vacuum tubes, which were slightly agitated to prevent the blood from coagulating. One milliliter was pipetted from the tube and poured into a second tube containing 1 mL of a 1% sterile solution of sodium polyanethol sulfonate (Sigma Chemical Co., St. Louis, MO, USA) to deactivate complement proteins. The additional 1 mL was stored at −80°C until PCR processing.

### Real-time PCR procedures

First, DNA was extracted from the samples. To quantify the total bacterial load and the periodontopathogen *P. gingivalis,* a real-time quantitative PCR (qPCR) technique was carried out in a 25 μL reaction volume. The cycling conditions were: 95°C for 10 minutes, 40 cycles at 95°C for 15 seconds, and 60°C for 1 minute. In the negative control, the DNA sample was replaced by sterile Milli-Q water. The primer/probe sequences were: *P. gingivalis -* forward: ACCTTACCCGGGATTGAAATG; reverse : CAACCATGCAGCACCTACATAGAA; probe: ATGACTGATGGTGAAAACCGTCTTCCCTTC; reference strain: W83 and total bacterial load - forward: TGGAGCATGTGGTTTAATTCGA; reverse: TGCGGGACTTAACCCAACA; probe: CACGAGCTGACGACA(AG)CCATGCA; reference strain: *E. coli* ATCC-25922. Standard curves, using a known amount of each bacterial species (10^1^-10^8^ cells), were employed to convert the cycle threshold values (CT) into the number of bacterial cells in the samples.

### Bacterial culture procedures

Immediately after collection, blood samples were spread out onto agar plates in duplicate^6^ ^,^ ^9^. Tryptic Soy Agar (TSA), incubated for 2 days at 35°C, was used to determine total aerobic counts, while Schaedler Blood Agar (SBA), incubated for 5-7 days at 35°C in an anaerobic chamber, was used to determine total anaerobic counts. Bacterial colony-forming units (CFU) were counted by an automated colony-counting system by a single researcher.

### Statistical analysis

Bacteremia occurrence (presence and/or increase of bacterial levels in the blood) was determined according to dental scaling. In addition, bacteremia data was compared between the groups (gingivitis vs. periodontitis) and among the times of sampling (pre-and post-scaling). The association between oral and blood bacterial levels and between PD and CAL and bacteremia were statistically analyzed. The bacterial levels determined by both laboratorial techniques were compared.

The primary outcome was the antimicrobial effect of a single mouth rinse use. To check this effect on bacteremia, the levels of viable anaerobic and aerobic bacterial cells were compared between 0 (T0) and 2 (T1) minutes, 0 (T0) and 6 (T2) minutes, and 2 (T1) and 6 (T2) minutes. Bacterial levels at 2 and 6 minutes were compared among the volunteers who performed the pre-procedural rinse and those who did not. As a secondary analysis, similar comparisons were performed considering the qPCR results.

Mann-Whitney and Signal tests were used in the above-mentioned comparisons (p<0.05). Data from the intention-to-treat analysis did not differ from the *per* protocol analysis.

## Results

Among the 27 periodontitis and 26 gingivitis individuals who composed the final study population, 49% underwent a pre-procedural oral rinse. To evaluate the effect of the pre-procedural rinse on blood bacterial levels, differences between the mean values observed at T1-T2 and TO were calculated, considering both the qPCR and culture results. We compared the mean values of individuals who rinsed to those who had not rinsed. Pre-procedural rinsing did not affect the levels of bacteria in the blood samples of both periodontitis and gingivitis individuals (Table 1).

**Table 1:** Comparative differences of mean bacterial levels (total bacterial load) and *P. gingivalis* observed at T1 and T2 and mean bacterial levels observed at T0 between individuals who performed the pre-procedural rinse or not according to periodontal diagnosis

			Differences of mean bacterial levels (T1,T2) - TO			
			qPCR		Culture	
Periodontal diagnosis	Culture	Pre-procedural rinse	Total bacterial load	Levels of *P. gingivalis*	Aerobic bacterial	Anaerobic bacterial
			(Primer universal)	(qPCR)	levels	levels
Gingivitis	Negative	No	30,176.0	0.0	1.5	0.4
		Yes	-6,303.6	0.2	0.0	0.0
		p-valor	0.85	1.00	0.55	0.81
	Positive	No	-6,422.5	0.1	115.8	8.8
		Yes	4,643.4	0.9	10.8	5.3
		p-valor	0.15	0.39	0.64	0.51
Periodontitis	Negative	No	1,783.5	-1.8	0.9	0.0
		Yes	-6,4959.0	1.2	0.0	0.0
		p-valor	0.70	0.70	0.62	1.00
	Positive	No	3,316.4	576.1	41.2	2.6
		Yes	-9,538.3	0.1	152.9	49.0
		p-valor	0.41	0.32	0.83	0.85

Mann-Whitney test (pre-procedural rinse positive vs. negative). Results were considered statistically significant when p<0.05

Total bacterial levels and levels of *P. gingivalis* in blood samples were compared between the periodontal diagnoses (gingivitis vs. periodontitis). Gingivitis patients exhibited lower blood bacterial levels, as demonstrated by both culture and qPCR at all sampling times (TO, T1 and T2) (p<0.0001; Mann-Whitney test). The levels of *P. gingivalis* were determined by qPCR and differed between gingivitis and periodontitis patients only at T2 (0.3 from gingivitis and 512.5 from periodontitis samples) (Table 2).

**Table 2:** Levels of bacteria (mean) according to laboratorial technique (qPCR and culture) before (T0), 2 (T1) and 6 minutes (T2) after dental scaling within a given periodontal diagnosis

Laboratorial technique	Diagnosis	N	T0		T1		T2	
qPCR			Total bacterial load (primer universal)	Levels of *P gingivalis* (qPCR)	Total bacterial load (primer universal)	Levels of *P gingivalis* (qPCR)	Total bacterial load (primer universal)	Levels of *P. gingivalis* (qPCR)
	Gingivitis	27	7,353.2	0.2	45,512.4	0.9	48,372.7	0.3
	Periodontitis	27	18,898.9	0.5	76,442.3	0.6	71,172.1	512.5
Mann-Whitney (p-value)			**<0.0001**	0.38	**<0.0001**	0.0874	**<0.0001**	**0.0036**
Culture	Diagnosis	27	Aerobic bacterial levels	Anaerobic bacterial levels	Aerobic bacterial levels	Anaerobic bacterial levels	Aerobic bacterial levels	Anaerobic bacterial levels
	Gingivitis	27	7.4	0.6	23.7	6.6	100.7	5.7
	Periodontitis		113.8	142.3	521.5	744.5	467.5	782.4
Mann-Whitney (p-value)			**<0.0001**	**0.0046**	**<0.0001**	**0.0099**	**<0.0001**	**0.0035**

Mann-Whitney test (quantitative polymerase chain reaction - qPCR vs. culture for gingivitis and periodontitis). A difference was statistically significant when p<0.05

To identify whether there was a better time for sampling, we calculated the differences between T1 and T0 values and between T2 and T0 values. Therefore, we checked whether a progressive increase or decrease occurred over time. For T2, culture bacterial counts from gingivitis patients showed 93.3 more bacteria (CFU) than T0, indicating that 6 minutes after the beginning of dental scaling is the best time to identify bacteremia. At this same time, the culture results from periodontitis patients only showed differences regarding anaerobic bacteria. For *P. gingivalis,* the qPCR results did not reveal any differences among sampling times (Table 3).

**Table 3:** Mean difference between time samplings (T1-T0) and (T2-T0) for all monitored bacterial levels according to periodontal diagnosis and laboratorial techniques

Diagnosis	Laboratorial technique	Bacterial levels	Difference	Mean ± Standard deviation	Signal test (p-value)	Mann-Whitney (test p-value)
Gingivitis	qPCR	Total bacterial load (primer universal)	T1-T0	-1,840.8 ± 29,467.9	0.83	0.96
			T2-T0	1,019.4 ± 24,730.7	0.83	
		*Porphyromonas gingivalis*	T1-T0	0.6 ± 2.3	0.16	0.19
			T2 -T0	0.0 ± 1.2	1.00	
	Culture	Aerobic bacterial levels	T1-T0	16.3 ± 101.2	0.79	0.02
			T2-T0	93.3 ± 239.8	0.004	
		Anaerobic bacterial levels	T1-T0	6.0 ± 13.9	0.018	0.82
			T2-T0	5.1 ± 12.7	0.026	
Periodontitis	qPCR	Total bacterial load (primer universal)	T1-T0	-4,456.6 ± 57,744.6	0.20	0.52
			T2-T0	-9,726.8 ± 55,713.3	0.81	
		*Porphyromonas gingivalis*	T1-T0	0.1 ± 2.3	0.82	0.90
			T2-T0	512.0 ± 2,659.3	0.43	
	Culture	Aerobic bacterial levels	T1-T0	107.6 ± 353.5	0.09	0.27
			T2-T0	53.7 ± 213.1	0.65	
		Anaerobic	T1 -T0	2.2 ± 9.7	0.40	0.25
		bacterial levels	T2-T0	40.1 ± 192.0	0.03	

Signal test checked if the following differences, T1-T0 and T2-T0, were different from zero. Mann-Whitney test compared difference TITO with difference T2-T0. Results were considered statistically significant when p<0.05 (95% confidence interval)

PD and CAL were compared between positive and negative bacteremia individuals. For the whole study population, the mean values differed according to the occurrence of bacteremia (Mann-Whitney test; p=0.0028 for PD and p=0.0014 for CAL). PD and CAL were higher among individuals who exhibited positive blood samples in culture (PD 3.5 mm vs. 2.1 mm and CAL 3.4 mm vs. 1.1 mm). Within each periodontal diagnosis, we did not observe similar differences (p>0.05) (Table 4).

**Table 4:** Comparative mean periodontal clinical (periodontal pocket depth - PD and clinical attachment level - CAL) and microbiological parameters (total bacterial load and levels of *P gingivalis* in subgingival samples), between positive and negative bacteremia individuals, according to periodontal diagnosis. Data for the entire study population is also shown

Periodontal diagnosis	Bacteremia (blood samples culture)	Periodontal pocket depth -PD Mean ± Standard deviation	Clinical attachment level -CAL Mean ± Standard deviation
Gingivitis	Negative	1.9 ± 0.8	0.6 ± 0.7
	Positive	1.8 ± 0.5	1.0 ± 0.8
	p-value	0.87	0.27
Periodontitis	Negative	3.2 ± 1.3	3.7 ± 0.8
	Positive	4.3 ± 1.3	4.6 ± 2.4
	p-value	0.17	0.39
Total population	Negative	2.1 ± 1.0	1.1 ± 1.4
	Positive	3.5 ± 1.6	3.4 ± 2.7
	p-value	**0.0028**	**0.0014**
**Periodontal diagnosis**	**Bacteremia (blood samples culture)**	**Total bacterial load (qPCR - primer universal) Mean ± Standard deviation**	**Levels of** *Porphyromonas gingivalis* **(qPCR) Mean ± Standard deviation**
Gingivitis	Negative	867,152.4 ±1,267,836.5	121,653.7 ± 432,896.3
	Positive	927,803.2 ± 927,237.2	1.5 ± 3.1
	p-value	0.46	0.26
Periodontitis	Negative	163,445.2 ± 38,278.0	22,485.4 ± 38,942.6
	Positive	3,223,390.4 ± 6,323,937.5	150,671.0 ± 301,174.9
	p-value	**0.0044**	0.42
Total population	Negative	742,968.8 ± 1,175,869.3	104,153.4 ± 392,390.1
	Positive	2,436,331.9 ± 5,232,368.3	99,012.9 ± 252,898.2
	p-value	**0.005**	0.32

Mann-Whitney test (bacteremia positive vs. bacteremia negative). Results were considered statistically significant when p<0.05 (95% confidence interval).

We also investigated the relation between subgingival bacterial profiles (total bacterial load and *P. gingivalis*) and bacteremia. Individuals who experienced bacteremia showed more bacteria in subgingival samples (Mann-Whitney test; p<0.005). In addition, periodontitis individuals who were positive for bacteremia also showed higher total bacterial levels in the subgingival area (p=0.004). On the other hand, there was no association between levels of subgingival bacteria and bacteremia among gingivitis individuals. Isolated, the levels of *P. gingivalis* did not vary according to bacteremia occurrence (Table 4).

## Discussion

Bacteremia is the presence of viable microorganisms in the blood stream. In the presence of periodontal diseases, the damaged tissues contribute to bacterial dissemination from oral sites throughout the body, linking oral health to systemic health. Therefore, it seems relevant to study the relation of bacteremia to periodontal status and dental scaling. Further, few studies have investigated the controlling effect of the pre-procedural rinse on induced bacteremia.

Despite its usual spontaneous resolution, among systemically compromised individuals, bacteremia is the main cause of septic shock^3^. Regardless of their pathogenic potentials in the oral cavity, once colonized in the extra-oral sites, oral bacteria often become *bona fide* pathogens, especially in immune-compromised individuals, causing disease manifestation. Oral bacteria had been involved with many systemic conditions, such as respiratory tract infections, meningitis, and brain, lung, liver, and splenic abscesses^11^.

Dental scaling was associated with higher blood bacterial levels at both 2 (60,977.3) and 6 minutes (59,722.4), as revealed by qPCR. We observed a similar increase regarding total counts of aerobic and anaerobic viable bacteria (648.20 at 2 minutes and 678.20 at 6 minutes). According to Horliana, et al.^13^ (2014), approximately half (49.4%) of periodontal procedures induce bacteremia. Periodontal probing and dental scaling cause bacteremia by stimulating soft diseased tissues in the periodontal pocket^14^. Therefore, periodontal diseases are a predisposing factor for bacteremia due to the infectious environment of the periodontal pocket and the lack of epithelial integrity^16^. In this study, the relation between periodontal breakdown and bacteremia was confirmed. Patients with positive cultured blood samples showed worse periodontal clinical status (PD and CAL). In addition, periodontal diagnosis impacted the magnitude of bacteremia. Periodontitis patients showed higher blood bacterial levels (culture and qPCR) than gingivitis patients. Kinane, et al.^14^ (2005) also observed that the incidence and magnitude of bacteremia were significantly higher in periodontitis than in gingivitis patients. Moreover, 6 minutes after scaling, periodontitis patients also exhibited higher blood levels of *P. gingivalis.* After dental scaling, other authors found *P. gingivalis* to be amongst the more frequent periodontal bacterial species in cultivated blood samples^20^ ^,^ ^22^. Further, *P. gingivalis* DNA is frequently found in non-oral sites - such as atheromatous plaques - in patients with periodontitis, suggesting a translocation from oral sites^26^. In addition, studies support a role for *P. gingivalis-mediated* periodontal disease as a risk factor for several systemic diseases including diabetes, preterm birth, stroke, and atherosclerotic cardiovascular disease^12^.

Mechanical procedures, such as those tested in this study, are commonly used as part of periodontal therapy. In addition, periodontal diseases are associated with higher levels of subgingival bacteria^5^, which, based on this study, could influence bacteremia. Our findings demonstrated that patients who experienced bacteremia had higher bacterial levels in subgingival biofilm samples. This finding should be further investigated in future studies.

Bacteremia is a transitory event. Most positive blood samples are identified between 30 seconds and 5 minutes after dental scaling^27^, and no more than 30 minutes^30^. However, the time of sampling is still a critical aspect of bacteremia research. In this study, for gingivitis patients, 6 minutes was the ideal sampling time for identifying viable bacteria. This same sampling time was the best for identifying anaerobic viable bacteria among periodontitis individuals. On the other hand, the bacterial time of sampling had no influence on bacterial DNA (qPCR - total bacterial load and *P. gingivalis)* in both periodontal diagnoses. Therefore, this study failed to identify an ideal sampling time for both techniques and both periodontal diagnosis. The inclusion of a low number of times of sampling represents a limitation of the study.

Regardless of time, the levels of bacteria were higher when the samples were analyzed by qPCR. To identify bacteremia, Kinane, et al.^14^ (2005) combined two laboratorial techniques. Even though these authors used conventional PCR, cultures revealed the lowest bacteremia rate. Chang, et al.^4^ (2013) published a systematic review on the subject and pointed out that PCR is an appropriate research tool for studying bacteremia. However, Benitéz-Páez, et al.^2^ (2013) highlighted that, in induced bacteremia, researchers do not always find enough DNA for PCR amplification. In the study by Marin, et al.^19^ (2016) neither culture nor qPCR detected any type of bacteria in the blood samples, while Ratto-Tespestini, et al.^23^ (2016) did not observe any superiority by qPCR over culture. Based on this concept, when possible, it seems reasonable to combine cultures and molecular analyses to clarify unanswered questions. However, in this study, the combination of two techniques enabled the identification of total viable bacteria and total bacterial DNA, for *P. gingivalis* no PCR procedures were able to identify viable bacteria cells. This could be interpreted as another limitation of this study to be handled in the future.

Health care procedures, such as periodontal therapeutic scaling, can induce transitory bacteremia^13^ ^,^ ^14^. In dentistry, the use of antimicrobial mouthrinses has different aims, however, the number of studies evaluating the effectiveness of preprocedural rinses on bacteremia is scarce, especially when considering RCT design in periodontal research. Due to its well-known antimicrobial properties, 0.12% chlorhexidine is one of the most recommend pre-procedural mouthrinse^29^. Therefore, this study evaluated blood bacterial levels after dental scaling, according to pre-procedural rinse. Unfortunately, a single 0.12% chlorhexidine pre-procedural rinse failed to reduce bacteremia occurrence among periodontally diseased individuals. Similarly, DuVall, et al.^7^ (2013) and Maharaj, et al.^18^ (2012) did not observe a decrease in bacteremia rates using this same pre-procedural rinse. In the study by Maharaj, et al.^18^ (2012), patients were randomly assigned to 0.12% chlorhexidine rinse, systemic antibiotics, or control. The control group received no intervention before dental extraction. Similarly, this study did not intervene before dental scaling in the control group. Maharaj, et al.^18^ (2012) found no difference between the rinse and control groups regarding bacteremia. DuVall, et al.^7^ (2013) randomized their patients into mouthrinse, antibiotics, or control. In this particular study, the control group was given a placebo. There is no clear information as to whether the mentioned placebo was in the form of capsules or rinse. In this study, however, we decided not to use a placebo rinse because previous studies reported increased bacterial counts in cultures after a 15-day placebo use^6^ ^,^ ^9^. In future studies, we shall handle this limitation. On the other hand, with a higher concentration of chlorhexidine (0.2%), Tuna, et al.^28^ (2012) reported a reduction in the incidence of bacteremia following dental extraction. Among these last studies, only ours monitored periodontal status, which could have partially affected the observed results. Sahrmann, et al.^24^ (2015) also evaluated the periodontal clinical status of a study population composed of periodontitis patients submitted to periodontal instrumentation with water or PVP-iodine rinse. Oral borne bacteremia was observed in 53% of the control group and in 11% of the test group. Bacteremia significantly reduced after PVP-iodine use.

Based on this study, although it is relevant for cross-infection control^8^ ^,^ ^10^, pre-procedural rinsing showed limited clinical relevance for bacteremia control. Interestingly, the results from the daily use of mouthrinse can differ from those observed after a single use. An essential-oil mouthrinse used for 15 days reduced bacteremia after mastication in gingivitis individuals^6^ ^,^ ^9^. However, in periodontitis patients, subgingival irrigation with essential-oils combined with one-week rinsing was not enough to decrease bacteremia following quadrant scaling^20^. Therefore, it could be speculated that to reduce bacteremia, antimicrobials would be prescribed 15 days before dental scaling and possibly continued for the duration of the treatment. Specifically, a pre-procedural rinse should be done aiming to control aerosol contamination and intra-oral infection.

Dental professionals should be able to appropriately manage periodontal patients and recognize bacteremia. In periodontally compromised individuals, high rates of bacteremia before scaling indicated the occurrence of bacteremia associated with daily activities. Preprocedural rinse did not reduce, effectively, levels of bacteria in the blood. Other strategies should be adopted to reduce bacteremia. The prescription of mouthrinse as a preventive measure against bacteremia should be further investigated. An appropriate management of bacterial biofilm in the subgingival area could contribute to prevent bacteremia in periodontally diseased individuals. Pre-procedural rinse did not reduce, effectively, levels of bacteria in the blood. Other strategies should be adopted to reduce bacteremia in periodontally diseased individuals.

## Conclusions

Dental scaling induced bacteremia in both gingivitis and periodontitis. However, bacteremia increased as periodontal compromising increased. The magnitude of bacteremia was greater among periodontitis patients. In periodontally diseased patients, pre-procedural rinsing showed a discrete effect on bacteremia control.
